# Effectiveness of Topic-Based Chatbots on Mental Health Self-Care and Mental Well-Being: Randomized Controlled Trial

**DOI:** 10.2196/70436

**Published:** 2025-04-30

**Authors:** Alan C Y Tong, Kent T Y Wong, Wing W T Chung, Winnie W S Mak

**Affiliations:** 1 Department of Psychology The Chinese University of Hong Kong Hong Kong China (Hong Kong)

**Keywords:** chatbot intervention, mental health literacy, self-care, randomized controlled trial, digital health, mental well-being, artificial intelligence

## Abstract

**Background:**

The global surge in mental health challenges has placed unprecedented strain on health care systems, highlighting the need for scalable interventions to promote mental health self-care. Chatbots have emerged as promising tools by providing accessible, evidence-based support. While chatbots have shown promise in delivering mental health interventions, most studies have only focused on clinical populations and symptom reduction, leaving a critical gap in understanding their preventive potential for self-care and mental health literacy in the general population.

**Objective:**

This study evaluated the effectiveness of a rule-based, topic-specific chatbot intervention in improving self-care efficacy, mental health literacy, self-care intention, self-care behaviors, and mental well-being immediately after 10 days and 1 month of its use.

**Methods:**

A 2-arm, assessor-blinded randomized controlled trial was conducted. A total of 285 participants were randomly assigned to the chatbot intervention group (n*=*140) and a waitlist control group (n*=*145). The chatbot intervention consisted of 10 topic-specific sessions targeting stress management, emotion regulation, and value clarification, delivered over 10 days with a 7-day free-access period. Primary outcomes included self-care self-efficacy, behavioral intentions, self-care behaviors, and mental health literacy. Secondary outcomes included depressive symptoms, anxiety symptoms, and mental well-being. Assessments were self-administered on the web at baseline, 10 days after the intervention, and at a 1-month follow-up. All outcomes were analyzed using linear mixed models with an intention-to-treat approach, and effect sizes were calculated using Cohen *d*.

**Results:**

Participants in the chatbot group demonstrated significantly greater improvements in behavioral intentions (*F*_2,379.74_=15.02; *P*<.001) and mental health literacy (*F*_2,423.57_=4.27; *P*=.02) compared to the control group. The chatbots were also able to bring significant improvement in self-care behaviors (Cohen *d=*0.36, 95% CI 0.08-0.30; *P*<.001), mindfulness (Cohen *d=*0.37, 95% CI 0.14-0.38; *P*<.001), depressive symptoms (Cohen *d=*–0.26, 95% CI –1.77 to –0.26; *P*=.004), overall well-being (Cohen *d=*0.22, 95% CI 0.02-0.42; *P*=.02), and positive emotions (Cohen *d=*0.28, 95% CI 0.08-0.54; *P*=.004) after 10 days. However, these improvements did not differ significantly at 1 month when compared to the waitlist control group. Adherence was higher among participants who received push notifications (*t*_138_=–4.91; *P*<.001).

**Conclusions:**

This study highlights the potential of rule-based chatbots in promoting mental health literacy and fostering short-term self-care intentions. However, the lack of sustained effects points to the necessary improvements required in chatbot design, including greater personalization and interactive features to enhance self-efficacy and long-term mental health outcomes. Future research should explore hybrid approaches that combine rule-based and generative artificial intelligence systems to optimize intervention effectiveness.

**Trial Registration:**

ClinicalTrials.gov NCT05694507; https://clinicaltrials.gov/ct2/show/NCT05694507

## Introduction

### Background

According to the World Health Organization [1], the COVID-19 pandemic caused mental health services to collapse in 93% of countries worldwide, given the extreme demand [2] in the face of the fragility of lives, economic downturn, sociopolitical tension, and conflicts [3]. Such extreme shortage of services prompted researchers, governments, and service agencies to develop viable options that can promote mental health self-care in the general population, prevent the worsening of mental health challenges, and promote overall mental health well-being, so that populations as a whole can be more resilient in facing future challenges rather than relying solely on a reactive, remedial treatment model that focuses on alleviating distress but falls short on promoting public mental health [4].

In Hong Kong, the public health care system faces similar challenges, with marked delays for the great majority of people seeking mental health services for common mental health disorders. The public psychiatry outpatient clinics documented more than 52,426 new bookings in the year 2023, with the longest waiting time reaching a concerning 100 weeks for people with relatively stable mental health conditions [5]. This delay is partly attributed to the current service model that relies on the physicians’ referral for appointment-based psychiatric consultations, where priority is given to people with severe mental illnesses. This causes most service seekers to queue up for their first consultation, with hardly any bridging service offered while waiting. There are also very limited alternatives other than private practices, which may not be affordable for most people.

Under such circumstances, chatbot technology offers new opportunities to bridge the gap by promoting mental health self-care [6]. Chatbots are computer programs designed to engage users through visual, spoken, or written language [7,8]. They are highly accessible and scalable as they provide around the clock support at people’s convenience, overcoming the limitations of traditional appointment-based therapy that is often offered only during working hours [9]. Interacting with a chatbot also reduces social pressure when disclosing sensitive and emotional issues, providing users with a confidential and nonjudgmental environment [10-12]. While some might question the loss of human touch with these virtual agents, Park et al [13] demonstrated that chatbots created with humanlike characteristics and interaction styles can elicit cooperation and trust from users, boosting the effectiveness of mental health therapies. Bickmore et al [14] also showed that individuals could develop therapeutic bonds with chatbots just as well as with human therapists, and sometimes prefer them over humans [15].

Currently, the 2 main types of mental health chatbots documented in the literature are rule-based chatbots, which use predefined rules or decision trees, and generative artificial intelligence (Gen AI) chatbots, which use machine learning and natural language processing. Abd-Alrazaq et al [10] identified that 89% of the published studies on mental health chatbots used the rule-based approach due to its suitability for handling simple, straightforward, and well-structured tasks. The controllability of using predefined responses reduces the likelihood of unforeseeable errors or harmful responses and ensures the evidence base of responses.

Evidence on chatbot-based interventions for mental health has steadily grown, although the findings remain mixed. Early studies suggested that chatbots can serve as an effective medium for delivering mental health interventions. For instance, Fitzpatrick et al [16] compared a cognitive behavioral therapy–oriented chatbot (Woebot) with an information-only control group and found that participants using Woebot showed significantly greater reduction in depressive symptoms (Cohen *d*=0.44), whereas those in the control group did not. Inkster et al [17] examined the mobile chatbot app Wysa using real-world data and compared high-use and low-use users and also found that high-usage users experienced notably greater reductions in depressive symptoms (Cohen *d*=0.47).

A recent meta-analysis by Zhong et al [18], encompassing 18 trials, identified moderate reduction in depression (*g*=–0.26) and anxiety (*g*=–0.19) after 8 weeks of treatment, although these effects did not persist at the 3-month follow-up. Meanwhile, Li et al [19] found a larger effect size in reducing depressive symptoms (*g*=0.64) and distress (*g*=0.70), but these same interventions did not enhance psychological well-being (*g*=0.32). On top of these mixed findings, studies exploring chatbot interventions are often hampered by small sample sizes or nonrandomized designs. In addition, many interventions focus narrowly on symptom alleviation rather than broader prevention work.

Recognizing the prevention potential of chatbots requires a shift in focus from merely symptom management to cultivating personal assets that empower individuals to engage in self-care behaviors. Self-efficacy, as described by Bandura [20], is a key determinant of health behavior change. Research has shown that higher self-efficacy is associated with greater engagement in health-promoting behaviors [21,22]. Enhancing self-efficacy is also suggested to be an effective strategy in mental health promotion [23]. Within the context of recovery and rehabilitation, self-care self-efficacy, that is, one’s perceived ability to perform relevant self-care activities, including strategies on relaxation, visualization, positive reframing, maintaining a positive attitude, and enjoying life [24], has been shown to relate positively with better adjustment in various populations, including people with cancer [25], people who have experienced stroke [26], and people with renal disease receiving hemodialysis [27]. Mak et al [28] showed that self-care self-efficacy substantially mediates the effect between social support and psychological adjustment, highlighting the important role of self-care self-efficacy in one’s mental health.

Concurrently, mental health literacy is another facilitator of self-care behaviors [29]. Mental health literacy is the knowledge and beliefs about mental disorders that aid in their recognition, management, or prevention [30]. Mental health literacy enables the critical evaluation of incoming information, facilitating individuals to make informed choices and avoid misinformation [31]. Choi [32] demonstrated the linkage between literacy, self-efficacy, and health-promoting behaviors, such that self-efficacy increases when an individual becomes more capable of making informed choices. This enhanced efficacy reinforces the uptake of health-promoting practices. Studies have shown that mental health literacy is crucial in encouraging early intervention and effective self-management, ultimately leading to better mental health outcomes [33,34].

### Objectives

Using a randomized controlled trial design, this study evaluated the effectiveness of a chatbot-based intervention (chatbot group) compared with a waitlist control group in improving self-help self-efficacy, mental health literacy, self-care intention, and the actual uptake of self-care behaviors. We had 3 hypotheses:

1. Participants in the chatbot group will experience superior changes in all the aforementioned aspects compared to the participants in the waitlist control group.
2. Chatbot group participants will also show superior improvement in mental health outcomes in terms of depressive and anxiety symptoms and mental well-being.
3. Participants who receive push notifications, compared to those who do not, will have better adherence in terms of higher completion rate of the chatbots.

## Methods

### Study Design

The study is a 2-arm, assessor-blinded randomized controlled trial comparing the effectiveness of a chatbot-based intervention with a waitlist control group. This study was preregistered on ClinicalTrials.gov (NCT05694507).

### Ethical Considerations

The trial protocol was approved by the Survey and Behavioral Research Ethics Committee of the Chinese University of Hong Kong (SBRE-22-0383). Before enrollment, all participants gave their informed consent on a web-based platform (Qualtrics) with their digital signature. They were informed about the study information and their rights to opt out or withdraw at the time of their choice without any consequence. The data were anonymized so that participants could not be identified. A compensation of HKD 50 (US $6.50) in payment voucher was offered to the participants for each valid completion of the study questionnaire.

### Participants

Participants included were (1) aged ≥18 years, (2) able to read and understand Chinese and spoken Cantonese, and (3) able to access the internet. As the contents of chatbots were extracted from an existing web-based mental well-being self-care platform, Jockey Club TourHeart+ Project [35], existing users of the platform and people who had participated in related research studies were excluded to avoid contamination. They were being batch-recruited through advertising on social media platforms (eg, Facebook and Instagram [Meta Platforms, Inc]), mass mailing at investigator’s institutions, and snowball sampling across 2 batches.

Upon registration, registrants gave informed consent on the web and were invited to complete a set of baseline questionnaires on a web-based surveying platform (Qualtrics; Qualtrics International Inc). The questionnaire was self-administered. A validation check, including validation items and time spent, was applied to confirm the quality of responses. Only those who gave valid responses to the baseline questionnaire were considered as valid participants in this study.

### Randomization and Blinding

Participants were randomized at a 1:1 ratio (chatbot vs waitlist control) using a computer-generated sequence created independently by the platform’s computer engineer. The randomization algorithm was validated before the study launch by an external data manager, who confirmed the sequence was free from identifiable patterns. To maintain concealment, neither the principal investigators nor the research staff had access to the algorithm or allocation list, which was stored on a secure server with restricted permissions. The blinding of participants was not feasible due to the nature of the intervention, but the research team remained unaware of group assignments until the study concluded. All allocation logs were locked and periodically audited to ensure no tampering occurred. Data analysts who conducted the final analyses were masked to participant identities throughout.

### Chatbot

Ten topic-specific chatbots covered a variety of topics, including relationships, stress management, value clarification, and emotion regulation, and used prevailing methods that have evidence in promoting mental health self-care and mental well-being, such as behavioral activation and positive psychology. In addition to psychoeducation about the topic, each chatbot also embedded related exercises, including mindfulness breathing and muscle relaxation practices. The materials in the chatbots were introduced by the virtual assistant called Boon, in Cantonese Chinese, the local dialect used in Hong Kong. It used person-centered language to make the conversations more humanlike with a personal touch. All contents and their rule-based responses were created by a team composed of a clinical psychology professor, a team of registered clinical psychologists, and psychological well-being officers, who are bachelor-level workers trained to provide low-intensity psychological interventions to individuals with common mental disorders. The 10 chatbot topics included the following:

1. How to communicate with others from the heart, with mindfulness practice
2. When anger knocks on your door—react or respond, with breathing and sensation practices
3. Stress—a motivator or an obstacle
4. Stress as our roommate, with mindfulness and muscle relaxation practices
5. Are you living the life you want to live?
6. Giving thoughts to your life—values and goals
7. Practicing self-care while taking care of others, with breathing practices
8. Emotion regulation with “ABC PLEASE” (A: accumulate positive emotions; B: build mastery; C: cope ahead; PL: treat physical illnesses; E: balance eating; A: avoid mood-altering substances; S: balance sleep; E: get exercise)
9. Loneliness versus solitude
10. Noticing beautiful things in life

**Figure 1:**
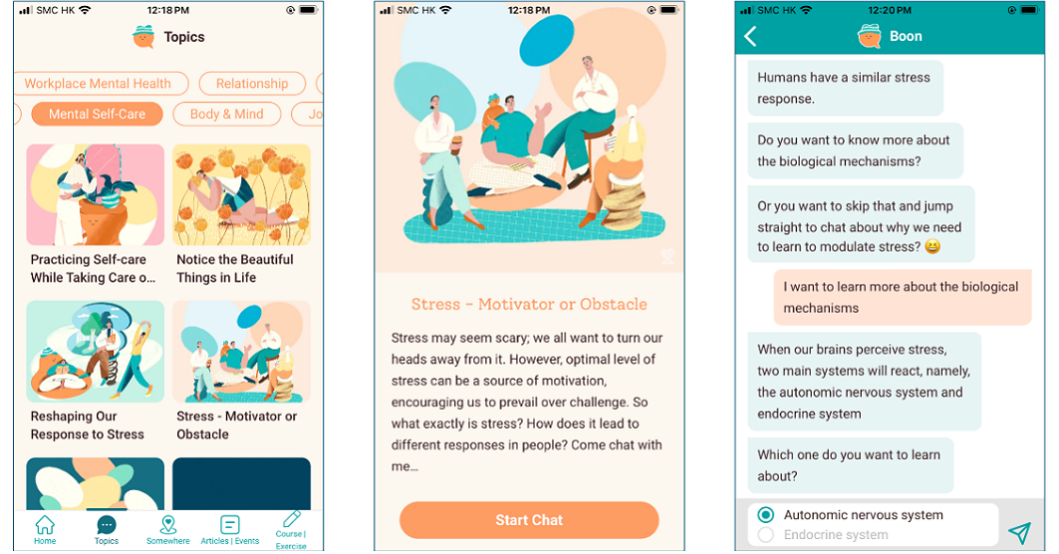
Screenshots of chatbots.

Participants interacted with these chatbots using predesigned categorical choice options. Each time the user clicked a chatbot, it welcomed the user and started the conversation. The chat progressed in turn-taking fashion between the chatbot and the user and new response options were presented after each response was made by the chatbot. Screenshots of chatbots are presented in Figure 1. Each chatbot took approximately 10 to 15 minutes to complete the entire episode of conversation. Whenever premature termination of the chatbot was detected, it asked whether the user would like to continue the same topic that they had left unfinished when they logged in again.

### Procedure

Upon enrollment, participants registered their accounts free of charge using a valid email on a web-based platform that contains the chatbots. Participants in the chatbot group completed one topic-specific chatbot per day for 10 consecutive days in the experimental phase, followed by a 7-day free-access period to the chatbots they had previously completed. During the experimental phase (the first 10 days), the 10 chatbots were distributed to each participant in random order, accounting for practice effect due to the specific sequence. Participants were required to complete the chatbot within the same day of distribution. Access to the chatbot expired the day after distribution, and participants were unable to revisit the same chatbot thereafter. During the subsequent free-access period, participants could freely browse through the chatbots they had completed previously for revision. However, any chatbots that participants did not complete during the experimental phase were not accessible during the free-access period.

Concurrently, participants in the waitlist control group did not have access to the chatbots until the end of the study. Upon completion of the study, all participants, including the ones from the waitlist control group, were granted access to all chatbots migrated to an integrated digital mental health platform, Jockey Club TourHeart+ [35].

All participants were invited to complete 3 questionnaires: baseline before randomization, on day 11 (after the test), and day 21 (follow-up) after group allocation. A validation check was applied to these questionnaires to ensure the quality of responses. A valid response was defined as >80% correctness on the attentional check items and within 2 SDs of average completion time. Participants were encouraged to inform researchers of any discomfort or adverse events caused by the chatbot at any time during the study.

### Deviations From the Registered Protocol

In the preregistered protocol, participants were randomly assigned to conditions with or without push notifications based on computer-generated random digits. However, during the actual trial run, a coding error prevented the algorithm from functioning as intended. Consequently, we decided to remove push notifications for all participants in the first batch. After observing a low completion rate of the chatbot tasks among participants in this initial batch, we modified the procedure for subsequent participants. Specifically, all participants recruited from the second batch received daily email reminders for 10 consecutive days following group allocation. These reminders served to notify participants about upcoming questionnaires, and for those in the chatbot group, each reminder included an additional link directing them to the chatbot.

### Measurements

This study adopted the Chinese version of all measurements. If there was no published Chinese version, standard translation, back-translation, and verification were conducted [36]. Information about participants, such as age, gender, education level, marital status, monthly income, occupational background, religion, experience in mental health–related services, and mindfulness practice, were included in the demographics section. Use data regarding the initiation and completion rates of chatbots were recorded in the backend system.

### Primary Outcomes

#### Self-Care Self-Efficacy

21 items from the stress reduction and positive attitudes subscales of the Strategies Used by People to Promote Health (SUPPH) [24] were used to assess the participants’ efficacy in carrying out self-care behaviors. SUPPH has been administered as a web-based questionnaire in recent digital-based studies [37,38], demonstrating its validity for online use. Items are measured on a 6-point scale from 1 (very little confidence) to 5 (quite a lot of confidence), with higher scores reflecting higher levels of self-care efficacy. The Cronbach α of these 21 items in this study was 0.94 at baseline, 0.95 after the survey, and 0.96 at follow-up.

#### Behavioral Intention

The behavioral intention subscale in the e-Therapy Attitude and Process Questionnaire (eTAP) [39] were adopted to measure participants’ perceived likelihood to execute self-care behaviors using digital means in the coming week. eTAP was originally designed as a web-based questionnaire, making it particularly suitable for assessing attitudes and intentions in digital intervention contexts. It includes 3 items, such as “it is likely I will use the web-based mental well-being intervention in the next week,” on a 7-point scale from 1 (strongly disagree) to 7 (strongly agree). The Cronbach α of these 3 items in this study was 0.97 at baseline, 0.97 after the survey, and 0.98 at follow-up.

#### Self-Care Behaviors

The 19-item Self-Care Behavior Inventory (SCBI) [40] was used to measure the uptake of self-care behaviors on a 5-point scale from 1 (very little) to 5 (quite a lot). Sample items include “exercise” and “eat healthily.” In this study, 1 item regarding medication was removed, and 2 self-constructed items related to time spent on things that respondents enjoy and feel interested in and time spent alone were added, resulting in 20 items in measuring actual self-care behaviors. Previous studies have successfully administered SCBI on the web [41], supporting its feasibility and reliability in digital survey formats. These 20 items had Cronbach α as 0.88 at baseline, 0.92 after the survey, and 0.92 at follow-up.

#### Mental Health Literacy

16 self-constructed items were used to measure the knowledge of various aspects related to mental health. These items were designed based on the taught materials of chatbots and the Mental Health Literacy Scale [42]. Sample items include “discerning the relationship between physical sensations, thoughts, behaviors, and emotions can help in managing emotions” and “emotional distress can be improved by reshaping responses habit towards distress.” Participants rated on a 7-point scale from 1 (strongly disagree) to 7 (strongly agree), with higher total scores reflecting better mental health literacy. One item, “emotions are a kind of ‘intuition’ that is difficult to discern,” was removed from the analysis to improve reliability. The Cronbach α of the 15 items included was 0.66 at baseline, achieved 0.77 after the test, and 0.76 at follow-up.

### Secondary Outcomes

#### Depressive Symptoms

The Patient Health Questionnaire (PHQ-9) [43] was used. It consists of 9 items assessing the extent to which respondents experience depression-related symptoms using a 4-point scale from 0 (not at all) to 3 (nearly every day). PHQ-9 has been validated and widely used in the general population for screening and measuring depression severity. Scores of 5, 10, 15, and 20 denote mild, moderate, moderately severe, and severe levels of depression, respectively (range 0-27). PHQ-9 has been available and validated as a web-based assessment in the Chinese population [44,45]. The Cronbach α of PHQ-9 in this study was 0.85 at baseline, 0.87 after the survey, and 0.88 at follow-up.

#### Anxiety Symptoms

The Generalized Anxiety Disorder Assessment–7 (GAD-7) [46] was used. It is a 7-item scale assessing the extent to which respondents experience anxiety-related symptoms using a 4-point scale from 0 (not at all) to 3 (nearly every day). Sample items included “feeling nervous, anxious, or on edge” and “worrying too much about different things.” Scores of 5, 10, and 15 denote the mild, moderate, and severe levels of anxiety, respectively (range 0-21). Similar to PHQ-9, web-based GAD-7 has been validated in the Chinese population [45,47]. The Cronbach α of the GAD-7 in this study was 0.92 at baseline, 0.90 after the survey, and 0.93 at follow-up.

#### Mental Well-Being

The Positive Emotion, Engagement, Relationships, Meaning, and Accomplishment (PERMA)–profiler [48] was used. It includes 23 items assessing various aspects of respondents’ well-being using an 11-point scale from 0 (never, terrible, or not at all) to 10 (always, excellent, or completely). Sample items include “in general, how often do you feel angry?” and “to what extent do you feel loved.” The PERMA subscales measure overall well-being, positive emotions, engagement, relationships, meaning, accomplishment, negative emotions, health, loneliness, and happiness. PERMA was introduced as an online assessment [48] and has been validated in the Chinese population [49]. In this study, these 23 items had good internal consistency with Cronbach α, which was 0.87 at baseline, 0.88 after the survey, and 0.89 at follow-up.

### Sample Size Estimation

Given there was no previous study on chatbot intervention targeting self-care behaviors, the sample size was estimated based on a generic effect size of small effect (*f*=0.1). Calculated using G*Power (version 3.0), 222 participants (74 per group) were required to detect a small within-between interaction effect of *f*=0.1 on a repeated measure ANOVA (90% power, significance=0.05, 3 groups, 4 measurements, assuming correlation among repeated measures=0.5, ε=1). This number was inflated to a planned sample size of 278 (139 per group) to account for an estimated 25% follow-up attrition rate.

### Statistical Analysis

All analyses were performed using SPSS (version 29; IBM Corp) [50]. ANOVA and *χ^2^* analysis were used on demographic variables (eg, age, gender, education, and baseline mental health variables) to examine differences between groups. Primary outcomes of self-care self-efficacy (SUPPH), behavioral intention (eTAP), self-care behaviors (SCBI), and mental health literacy (Mental Health Literacy Scale) and secondary outcomes of depressive symptoms (PHQ-9), anxiety symptoms (GAD-7), and mental well-being (PERMA) were analyzed using linear mixed models (LMMs) with an intention-to-treat approach. Mixed models were a well-established method for imputation of missing values using restricted maximum likelihood [51].

In the LMM, random intercept models were fitted with intercepts included for each individual. The fixed effects included group (chatbot group or waitlist control group), time (baseline, after 10 days, and 1-month follow-up evaluation), and their associated interaction effects. Since receiving push notifications has a significant effect on adherence, it was included as a covariate in the LMM analyses. To follow up on any significant interaction effect (2-way time × group interaction), post hoc pairwise comparisons with Bonferroni adjustment were conducted by looking into the effects fixing at each level of time and condition. Pre-post and pre–follow-up repeated measures effect sizes (Cohen dRM) were calculated following the study by Du et al [44]. Between-group effects and interaction effect sizes were calculated using a web-based calculator, Psychometrica—form 2 and 3, respectively [52]. Effect sizes were interpreted according to Cohen guidelines, where 0.2 indicates a small effect, 0.5 a medium effect, and 0.8 a strong effect.

As for sensitivity testing, another series of per-protocol analyses were conducted using the same procedure as mentioned earlier. Per-protocol criteria were defined as the completion of all assessments without missing data and completing at least 1 chatbot for those who were assigned to the chatbot group.

## Results

### Participant Characteristics

**Figure 2:**
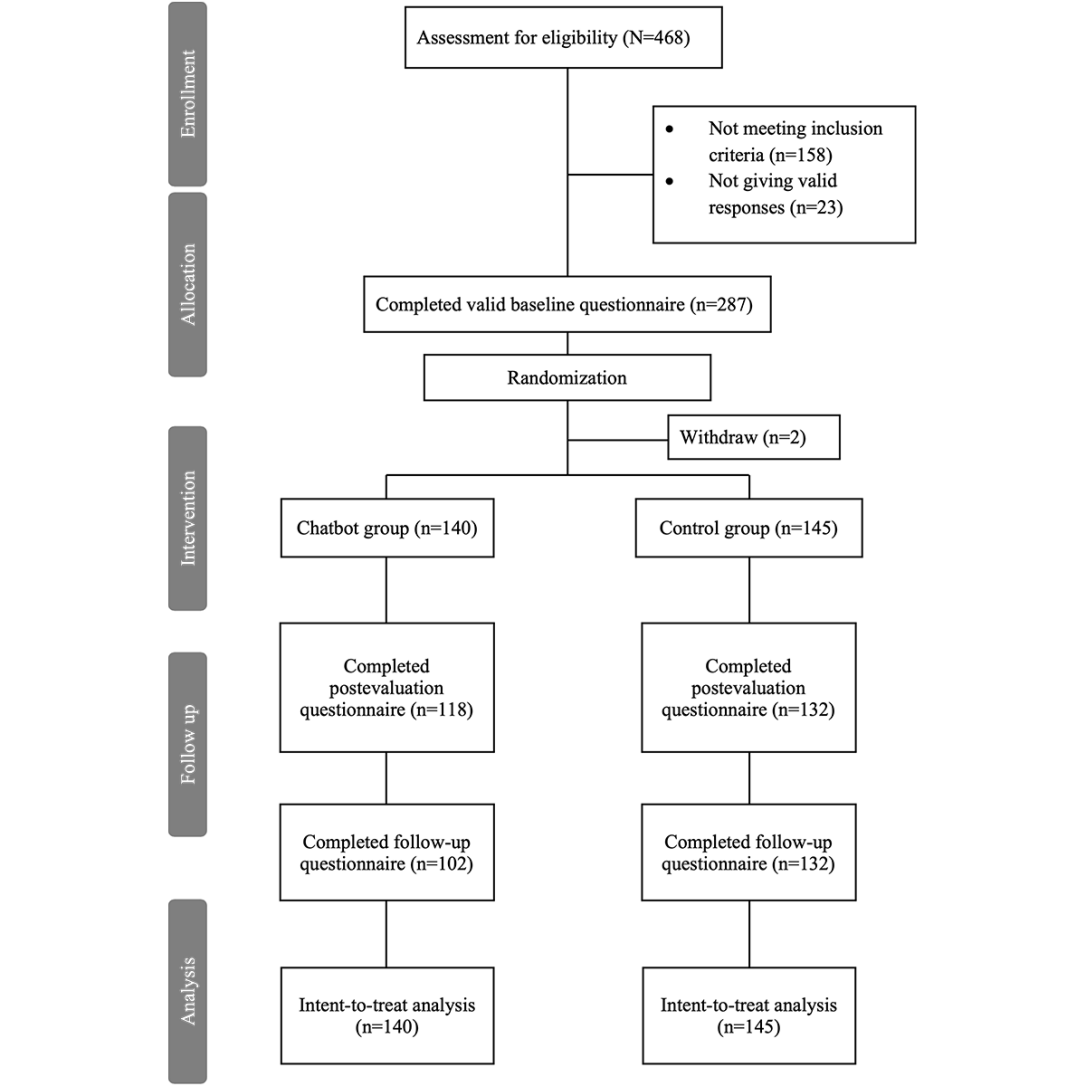
CONSORT (Consolidated Standards of Reporting Trials) diagram of study flow.

Participants were recruited between January 2023 and March 2023. A total of 468 individuals registered for the study across 2 batches, gave informed consent, and completed the screening survey to check for their eligibility. In total, 33.8% (158/468) of the participants did not meet the inclusion criteria, and 4.9% (23/468) of them provided invalid responses on the baseline survey and were excluded. In total, 0.4% (2/468) of participants withdrew afterward before the commencement of intervention due to personal reasons and were further excluded. The final intention-to-treat sample consisted of 285 individuals with 140 individuals being randomized to the chatbot group and 145 in the waitlist control group. Figure 2 provides the CONSORT (Consolidated Standards of Reporting Trials) flowchart, while the CONSORT-EHEALTH checklist is included as Multimedia Appendix 1. Results of *t* tests (2-tailed) and *χ*^2^ tests showed no significant differences in the demographics and outcomes between the chatbot and control groups and between those who received and did not receive notifications. Table 1 summarizes the demographic characteristics of the participants.

**Table 1:** Participant characteristics.

Characteristics			Whole sample (n*=*285)		Chatbot group (n*=*140)		Waitlist control group (n*=*145)
Age (y), mean (SD); range			26.45 (8.37); 18-61		26.15 (8.02); 18-58		26.73 (8.71); 18-61
**Gender, n (%)**							
	Men	68 (23.9)		33 (23.6)		35 (24.1)	
	Women	216 (75.8)		107 (76.4)		109 (75.2)	
**Education level, n (%)**							
	Secondary or lower	27 (9.5)		11 (7.9)		16 (11)	
	Diploma, certificate, or associate degree	9 (3.2)		6 (4.3)		3 (2.1)	
	Bachelor degree	177 (62.1)		89 (63.6)		88 (60.7)	
	Master degree	60 (21.1)		28 (20)		32 (22.1)	
	Doctoral degree	12 (4.2)		6 (4.3)		6 (4.1)	
**Religion, n (%)**							
	No religion	214 (75.1)		100 (71.4)		114 (78.6)	
	Buddhism	9 (3.2)		4 (2.9)		5 (3.4)	
	Taoism	2 (0.7)		0 (0)		2 (1.4)	
	Christianity	48 (16.8)		30 (21.4)		18 (12.4)	
	Catholicism	12 (4.2)		6 (4.3)		6 (4.1)	
**Marital status, n (%)**							
	Single	198 (69.5)		98 (70)		100 (69)	
	Unmarried	45 (15.8)		26 (18.6)		19 (13.1)	
	Cohabited	8 (2.8)		3 (2.1)		5 (3.4)	
	Married	33 (11.6)		13 (9.3)		20 (13.8)	
	Divorced or separated	1 (0.4)		0 (0)		1 (0.7)	
**Monthly income (HKD^a^), n (%)**							
	<10,000	107 (37.5)		60 (42.9)		47 (32.4)	
	10,000-29,999	82 (28.8)		40 (28.6)		42 (29)	
	30,000-59,999	36 (12.6)		18 (12.9)		18 (12.4)	
	>60,000	8 (2.8)		4 (2.9)		4 (2.8)	
	Others or not disclosed	52 (18.2)		18 (12.9)		34 (23.4)	
**Counseling service or psychotherapy, n (%)**							
	Received in the past	28 (9.8)		15 (10.7)		13 (9)	
	Currently receiving	17 (6)		7 (5)		10 (6.9)	
	None	240 (84.2)		118 (84.3)		122 (84.1)	
**Other web-based self-guided psychological intervention, n (%)**							
	Used in the past	10 (3.5)		6 (4.3)		4 (2.8)	
	None	275 (96.5)		134 (95.7)		141 (97.2)	

^a^The average conversion rate during the study was 1 HKD=US $0.13.

### Attrition Analysis

Among the 285 participants, 35 (12.3%) of them dropped out after the assessment, and 51 (17.9%) dropped out at 1-month follow-up. Significantly more participants dropped out in the chatbot group than in the control group at follow-up (*χ*^2^_1_=16, *P*<.001). Their reasons for withdrawal were unknown since we did not administer an exit survey. No instance of serious adverse events was reported throughout the study period. Notification status (received or not received) had no significant effect on attrition after the evaluation (*χ*^2^_1_=0.1, *P*=.71) and at follow-up (*χ*^2^_1_=3.3, *P*=.07).

Mean baseline depressive and anxiety symptoms were significantly higher among those who dropped out after the test (PHQ-9: mean 9.17, SD 5.16; GAD-7: mean 10.14, SD 5.70) than those who stayed (PHQ-9: mean 7.25, SD 5.09; GAD-7: mean 7.73, SD 6.05; PHQ-9: *t*_283_=2.09, *P*=.04; GAD-7: *t*_283_=2.23, *P*=.03). Their overall mental well-being (mean 5.46, SD 1.55), as assessed by the PERMA profiler, was also marginally lower than that of the retained participants (mean 6.11, SD 1.45; *t*_42.70_=–2.35; *P*=.01).

Similarly, those who dropped out at follow-up had significantly higher PHQ-9 scores (mean 9.57, SD 5.37) and higher GAD-7 scores (mean 11.22, SD 6.86) than those who stayed (PHQ-9: mean 7.03, SD 4.97; GAD-7: mean 7.33, SD 5.64; PHQ-9: *t*_283_=3.26, *P*=.001; GAD-7: *t*_283_=4.28, *P*<.001). These revealed that selective bias was presented, with retained participants having better initial mental health in terms of lower levels of depression and anxiety symptoms and higher levels of mental well-being.

### Chatbot Use

All the 140 participants in the chatbot group initiated at least one of the 10 topic-based chatbots during the first 10 days. 12 participants (8.6%) terminated the first chat prematurely and did not start another. Of 140, 128 (91.4%) finished at least one entire conversation. In total, 33.6% (47/140) of participants completed all 10 chatbots, and 14.3% (20/140) of them completed 9 chatbots. The remaining percentages were evenly distributed across the number of chatbots completed. On average, participants completed 6.78 chatbots (SD 3.50). Push notifications had a significant effect on the chatbot completion rate (*t*_138_=–4.91, *P*<.001). The 85 participants who received push notifications completed significantly more chatbots (mean 7.86, SD 3.30) than the 55 participants who did not receive notifications (mean 5.11, SD 3.14).

### Effects on Primary Outcomes

#### Overview

**Figure 3:**
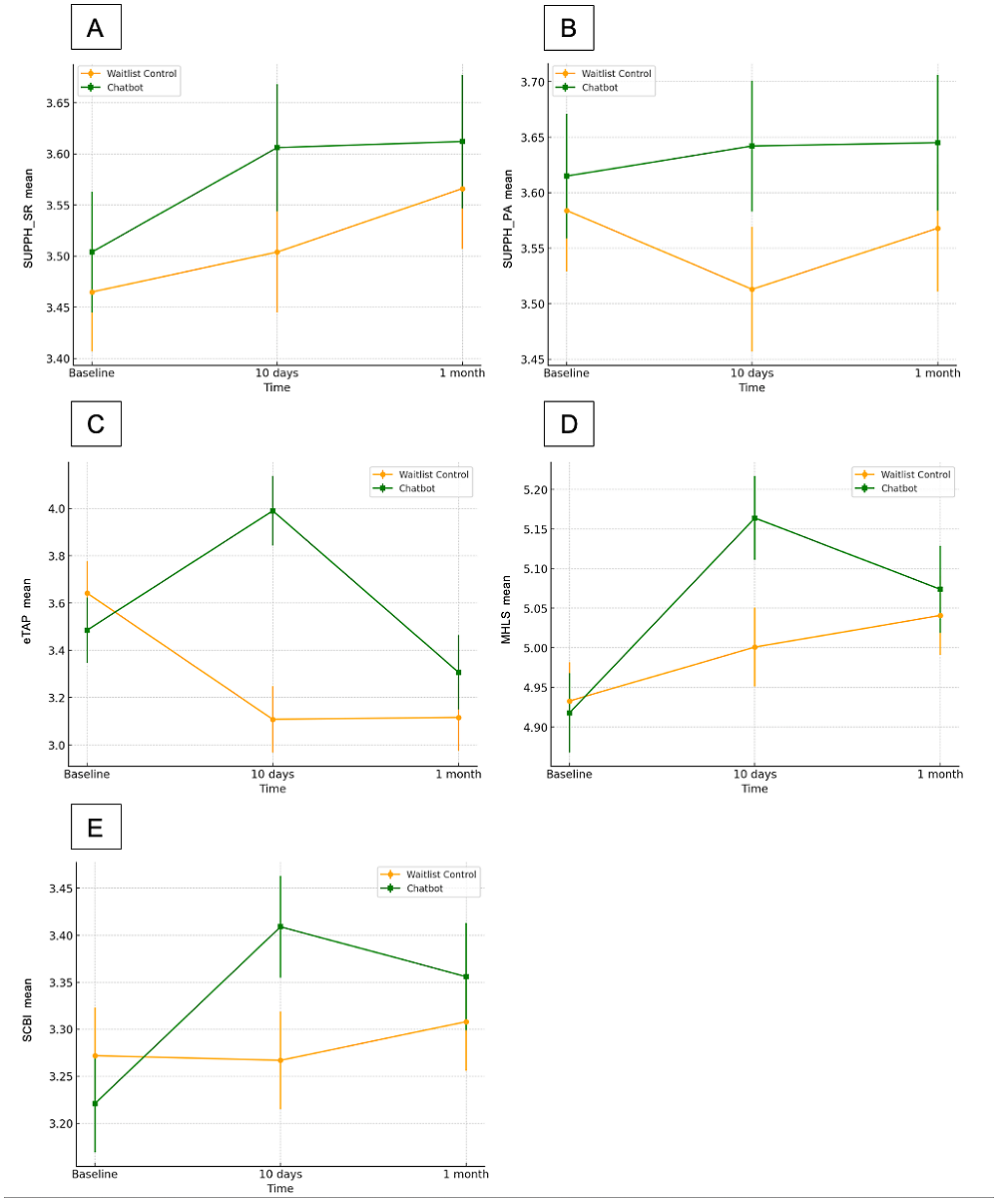
Changes in primary outcomes over time by condition. (A) Changes in Strategies Used by People to Promote Health (SUPPH) SUPPH_SR, (B) changes in SUPPH_PA, (C) changes in e-Therapy Attitude and Process Questionnaire (eTAP), (D) changes in Mental Health Literacy Scale (MHLS), (E) changes in Self-Care Behaviors Inventory (SCBI).

Table 2 shows the means and SDs, as well as the estimated marginal means and SEs in the LMM analyses of outcome variables measured at each time point. Tables 3 and 4 demonstrate the detailed statistics of within-group pairwise comparisons from baseline to 10 days and from baseline to 1 month respectively. Table 5 shows the detailed statistics of between-group pairwise comparisons. Figure 3 shows the changes in the primary outcomes overtime by condition graphically.

**Table 2:** Observed means and estimated marginal means of outcomes.

Variable (measurement)			Chatbot group														Waitlist control group													
			Baseline (n*=*140)				10 days (n*=*118)				1 month (n*=*102)					Baseline (n*=*145)					10 days (n*=*132)					1 month (n*=*132)				
			Values, mean (SD)		EMM^a^ (SE)		Values, mean (SD)		EMM (SE)		Values, mean (SD)		EMM (SE)		Values, mean (SD)				EMM (SE)		Values, mean (SD)		EMM (SE)		Values, mean (SD)			EMM (SE)		
**Self-care self-efficacy (SUPPH^b^)**																														
	Stress reduction	3.51 (0.72)		3.50 (0.06)		3.62 (0.66)		3.61 (0.06)		3.62 (0.65)		3.61 (0.07)		3.47 (0.66)				3.47 (0.06)		3.52 (0.74)		3.50 (0.06)		3.58 (0.69)			3.57 (0.06)			
	Positive attitudes	3.61 (0.61)		3.62 (0.06)		3.66 (0.64)		3.64 (0.06)		3.65 (0.60)		3.65 (0.06)		3.58 (0.66)				3.58 (0.06)		3.54 (0.70)		3.51 (0.06)		3.59 (0.73)			3.57 (0.06)			
Behavioral intention (eTAP^c^)			3.49 (1.72)		3.49 (0.14)		4.00 (1.61)		3.99 (0.15)		3.32 (1.59)		3.31 (0.16)		3.64 (1.69)				3.64 (0.14)		3.09 (1.51)		3.11 (0.14)		3.11 (1.58)			3.12 (0.14)		
Self-care behaviors (SCBI^d^)			3.22 (0.55)		3.22 (0.05)		3.44 (0.62)		3.41 (0.05)		3.37 (0.60)		3.36 (0.06)		3.27 (0.61)				3.27 (0.05)		3.28 (0.59)		3.27 (0.05)		3.32 (0.66)			3.31 (0.05)		
Mental health literacy (MHLS^e^)			4.92 (0.54)		4.92 (0.05)		5.19 (0.65)		5.16 (0.05)		5.07 (0.65)		5.07 (0.06)		4.93 (0.55)				4.93 (0.05)		5.03 (0.57)		5.00 (0.05)		5.06 (0.58)			5.04 (0.05)		
Depressive symptoms (PHQ-9^f^)			7.80 (4.77)		7.78 (0.44)		6.58 (4.69)		6.76 (0.45)		6.85 (4.82)		7.28 (0.47)		7.18 (5.45)				7.20 (0.43)		7.07 (5.52)		7.27 (0.44)		7.14 (5.30)			7.33 (0.44)		
Anxiety symptoms (GAD-7^g^)			8.34 (5.81)		8.32 (0.46)		6.71 (4.38)		6.94 (0.48)		6.40 (5.14)		7.03 (0.50)		7.72 (6.28)				7.73 (0.45)		6.92 (4.76)		7.13 (0.46)		6.73 (5.16)			6.87 (0.46)		
Mindfulness (MAAS^h^)			4.05 (0.79)		4.05 (0.07)		4.33 (0.72)		4.31 (0.07)		4.40 (0.71)		4.34 (0.07)		4.18 (0.80)				4.18 (0.07)		4.21 (0.81)		4.21 (0.07)		4.35 (0.79)			4.34 (0.07)		
**Well-being (PERMA^i^)**																														
	Overall well-being	5.97 (1.35)		5.97 (0.13)		6.28 (1.44)		6.19 (0.14)		6.76 (1.55)		6.67 (0.14)		6.09 (1.59)				6.09 (0.13)		6.1 (1.66)		6.04 (0.13)		6.66 (1.72)			6.62 (0.13)			
	Accomplishment	5.68 (1.75)		5.68 (0.15)		6.01 (1.72)		5.95 (0.16)		6.35 (1.81)		6.26 (0.17)		5.72 (1.78)				5.72 (0.15)		5.79 (1.85)		5.75 (0.15)		6.10 (1.93)			6.10 (0.16)			
	Positive emotion	6.05 (1.41)		6.05 (0.15)		6.47 (1.59)		6.36 (0.15)		6.99 (1.72)		6.86 (0.16)		6.22 (1.78)				6.22 (0.14)		6.16 (1.89)		6.08 (0.15)		6.96 (1.87)			6.91 (0.15)			
	Engagement	5.92 (1.58)		5.93 (0.14)		6.15 (1.54)		6.10 (0.15)		6.65 (1.58)		6.61 (0.16)		5.99 (1.69)				5.99 (0.14)		5.89 (1.75)		5.86 (0.14)		6.51 (1.83)			6.48 (0.14)			
	Meaning	5.79 (1.92)		5.79 (0.17)		6.11 (1.87)		6.05 (0.18)		6.38 (2.00)		6.29 (0.18)		5.83 (2.08)				5.83 (0.17)		5.92 (2.08)		5.88 (0.17)		6.23 (2.01)			6.22 (0.17)			
	Relationships	6.29 (1.62)		6.29 (0.16)		6.49 (1.78)		6.36 (0.16)		7.13 (1.85)		7.04 (0.17)		6.49 (1.91)				6.50 (0.15)		6.59 (1.85)		6.49 (0.16)		7.20 (1.95)			7.12 (0.16)			
	Negative emotion	5.11 (1.69)		5.10 (0.16)		4.73 (1.94)		4.72 (0.16)		5.12 (1.98)		5.19 (0.17)		4.76 (1.82)				4.76 (0.15)		4.67 (1.87)		4.69 (0.16)		5.14 (1.92)			5.14 (0.16)			
	Health	5.75 (1.91)		5.74 (0.17)		6.03 (1.96)		5.91 (0.17)		6.62 (2.01)		6.47 (0.18)		6.00 (1.86)				6.01 (0.16)		5.96 (1.9)		5.91 (0.16)		6.64 (2.02)			6.60 (0.17)			
	Loneliness	5.05 (2.28)		5.04 (0.20)		4.80 (2.35)		4.75 (0.21)		5.61 (2.47)		5.65 (0.22)		4.61 (2.42)				4.61 (0.20)		4.63 (2.45)		4.65 (0.21)		5.58 (2.50)			5.65 (0.21)			

^a^EMM: estimated marginal mean.

^b^SUPPH: Strategies Used by People to Promote Health.

^c^eTAP: e-Therapy Attitude and Process Questionnaire.

^d^SCBI: Self-Care Behaviors Inventory.

^e^MHLS: Mental Health Literacy Scale.

^f^PHQ-9: Patient Health Questionnaire.

^g^GAD-7: Generalized Anxiety Disorder Assessment–7.

^h^MAAS: Mindful Attention Awareness Scale.

^i^PERMA: Positive Emotion, Engagement, Relationships, Meaning, and Accomplishment.

**Table 3:** Pairwise comparisons of time effects from baseline to 10 days by condition with Bonferroni adjustment

Variable (measurement)		Chatbot group				Waitlist control group		
		Mdiff^a^ (95% CI)	*P* value	dRM^b^	Mdiff (95% CI)		*P* value	dRM
**Self-care self-efficacy (SUPPH^c^)**								
	Stress reduction	0.10 (–0.04 to 0.24)	.24	0.17	0.04 (–0.10 to 0.17)		.99	0.07
	Positive attitudes	0.03 (–0.09 to 0.14)	.99	0.08	–0.07 (–0.18 to 0.04)		.35	–0.07
Behavioral intention (eTAP^d^)		0.51 (0.16 to 0.85)	.001	0.31	–0.53 (–0.86 to –0.21)		<.001	–0.35
Self-care behaviors (SCBI^e^)		0.19 (0.08 to 0.30)	<.001	0.36	–0.01 (–0.11 to 0.10)		.99	0.02
Mental health literacy (MHLS^f^)		0.25 (0.14 to 0.35)	<.001	0.45	0.07 (–0.04 to 0.17)		.34	0.17
Depressive symptoms (PHQ-9^g^)		–1.02 (–1.77 to –0.26)	.004	–0.26	0.07 (–0.64 to 0.79)		.99	–0.02
Anxiety symptoms (GAD-7^h^)		–1.38 (–2.30 to –0.47)	<.001	–0.31	–0.60 (–1.47 to 0.27)		.30	–0.14
Mindfulness (MAAS^i^)		0.26 (0.14 to 0.38)	<.001	0.37	0.02 (–0.09 to 0.14)		.99	0.04
**Well-being (PERMA^j^)**								
	Overall well-being	0.22 (0.02 to 0.42)	.02	0.22	–0.05 (–0.24 to 0.13)		.99	<0.01
	Accomplishment	0.27 (–0.01 to 0.55)	.07	0.19	0.03 (–0.23 to 0.30)		.99	0.04
	Positive emotion	0.31 (0.08 to 0.54)	.004	0.28	–0.14 (–0.36 to 0.08)		.35	–0.03
	Engagement	0.18 (–0.12 to 0.48)	.46	0.15	–0.13 (–0.42 to 0.15)		.80	–0.06
	Meaning	0.27 (–0.02 to 0.56)	.08	0.17	0.05 (–0.22 to 0.33)		.99	0.05
	Relationships	0.07 (–0.19 to 0.33)	.99	0.12	–0.01 (–0.25 to 0.24)		.99	0.05
	Negative emotion	–0.38 (–0.69 to –0.07)	.01	–0.21	–0.07 (–0.37 to 0.22)		.99	–0.05
	Health	0.17 (–0.12 to 0.45)	.49	0.14	–0.09 (–0.36 to 0.18)		.99	–0.02
	Loneliness	–0.29 (–0.73 to 0.15)	.35	–0.11	0.03 (–0.38 to 0.45)		.99	0.01

^a^Mdiff: mean difference.

^b^dRM: repeated measure Cohen *d*.

^c^SUPPH: Strategies Used by People to Promote Health.

^d^eTAP: e-Therapy Attitude and Process Questionnaire.

^e^SCBI: Self-Care Behaviors Inventory.

^f^MHLS: Mental Health Literacy Scale.

^g^PHQ-9: Patient Health Questionnaire.

^h^GAD-7: Generalized Anxiety Disorder Assessment–7.

^i^MAAS: Mindful Attention Awareness Scale.

^j^PERMA: Positive Emotion, Engagement, Relationships, Meaning, and Accomplishment.

**Table 4:** Pairwise comparisons of time effects from baseline to 1 month by condition with Bonferroni adjustment

Variable (measurement)			Chatbot group							Waitlist control group				
			Mdiff^a^ (95% CI)		*P* value		dRM^b^		Mdiff (95% CI)			*P* value		dRM
**Self-care self-efficacy (SUPPH^c^)**														
	Stress reduction	0.11 (–0.03 to 0.25)		.20		0.17		0.10 (–0.03 to 0.23)			.17		0.18	
	Positive attitudes	0.03 (–0.09 to 0.15)		.99		0.06		–0.02 (–0.13 to 0.10)			.99		<0.01	
Behavioral intention (eTAP^d^)			–0.18 (–0.60 to 0.24)		.93		–0.10		–0.53 (–0.91 to –0.14)			.003		–0.32
Self-care behaviors (SCBI^e^)			0.14 (0.01 to 0.26)		.03		0.26		0.04 (–0.08 to 0.15)			.99		0.07
Mental health literacy (MHLS^f^)			0.16 (0.05 to 0.27)		.002		0.24		0.11 (0.01 to 0.21)			.03		0.22
Depressive symptoms (PHQ-9^g^)			–0.50 (–1.43 to 0.44)		.60		–0.19		0.14 (–0.71 to 0.98)			.99		–0.01
Anxiety symptoms (GAD-7^h^)			–1.30 (–2.34 to –0.25)		.009		–0.31		–0.87 (–1.81 to 0.08)			.09		–0.16
Mindfulness (MAAS^i^)			0.29 (0.16 to 0.42)		<.001		0.47		0.16 (0.04 to 0.28)			.005		0.22
**Well-being (PERMA^j^)**														
	Overall well-being	0.70 (0.46 to 0.94)		<.001		0.51		0.53 (0.31 to 0.75)			<.001		0.31	
	Accomplishment	0.58 (0.25 to 0.91)		<.001		0.36		0.38 (0.08 to 0.68)			.007		0.19	
	Positive emotion	0.81 (0.52 to 1.11)		<.001		0.55		0.69 (0.42 to 0.96)			<.001		0.42	
	Engagement	0.69 (0.36 to 1.02)		<.001		0.45		0.49 (0.20 to 0.79)			<.001		0.28	
	Meaning	0.51 (0.19 to 0.82)		<.001		0.28		0.39 (0.11 to 0.67)			.003		0.22	
	Relationships	0.75 (0.45 to 1.05)		<.001		0.47		0.62 (0.35 to 0.89)			<.001		0.33	
	Negative emotion	0.08 (–0.25 to 0.42)		.99		<0.01		0.37 (0.07 to 0.67)			.009		0.20	
	Health	0.73 (0.41 to 1.05)		<.001		0.43		0.59 (0.31 to 0.88)			<.001		0.30	
	Loneliness	0.61 (0.12 to 1.09)		.008		0.22		1.04 (0.60 to 1.48)			<.001		0.39	

^a^Mdiff: mean difference.

^b^dRM: repeated measure Cohen *d*.

^c^SUPPH: Strategies Used by People to Promote Health.

^d^eTAP: e-Therapy Attitude and Process Questionnaire.

^e^SCBI: Self-care Behaviors Inventory.

^f^MHLS: Mental Health Literacy Scale.

^g^PHQ-9: Patient Health Questionnaire.

^h^GAD-7: Generalized Anxiety Disorder Assessment–7.

^i^MAAS: Mindful Attention Awareness Scale.

^j^PERMA: Positive Emotion, Engagement, Relationships, Meaning, and Accomplishment.

**Table 5:** Pairwise comparisons of group effects by time point with Bonferroni adjustment.

Variable (measurement) and time			Mdiff^a^ (95% CI)	Group effect, *F* test (*df*)	*P* value	Between-group Cohen *d*	
**Self-care self-efficacy (SUPPH^b^)—stress reduction**							
	10 days		0.1 (–0.07 to 0.27)	1.43 (1, 515.16)	.23	0.15	
	1 month		0.05 (–0.13 to 0.22)	0.27 (1, 543.38)	.60	0.06	
**Self-care self-efficacy (SUPPH)—positive attitudes**							
	10 days		0.13 (–0.03 to 0.29)	2.5 (1, 455.12)	.12	0.19	
	1 month		0.08 (–0.09 to 0.24)	0.85 (1, 478.16)	.36	0.09	
**Behavioral intention (eTAP^c^)**							
	10 days		0.88 (0.49 to 1.28)	18.97 (1, 591.11)	<.001	0.59	
	1 month		0.19 (–0.22 to 0.61)	0.83 (1, 602.28)	.36	0.13	
**Self-care behaviors (SCBI^d^)**							
	10 days		0.14 (–0.01 to 0.29)	3.52 (1, 471.61)	.06	0.26	
	1 month		0.05 (–0.11 to 0.2)	0.38 (1, 497.46)	.54	0.08	
**Mental health literacy (MHLS^e^)**							
	10 days		0.16 (0.02 to 0.31)	5.02 (1, 470.52)	.03	0.27	
	1 month		0.03 (–0.11 to 0.18)	0.19 (1, 497.15)	.66	0.02	
**Depressive symptoms (PHQ-9^f^)**							
	10 days		–0.51 (–1.74 to 0.73)	0.66 (1, 414.13)	.42	0.09	
	1 month		–0.05 (–1.32 to 1.21)	0.01 (1, 438.99)	.94	0.06	
**Anxiety symptoms (GAD-7^g^)**							
	10 days		–0.20 (–1.5 to 1.11)	0.09 (1, 448.78)	.77	0.05	
	1 month		0.16 (–1.18 to 1.5)	0.06 (1, 468.61)	.82	0.06	
**Mindfulness (MAAS^h^)**							
	10 days		0.10 (–0.09 to 0.29)	1.07 (1, 411.32)	.30	0.16	
	1 month		–0.01 (–0.2 to 0.19)	0.004 (1, 432.10)	.95	0.06	
**Well-being (PERMA^i^)**							
	**Overall well-being**						
		10 days	0.15 (–0.22 to 0.53)	0.66 (1, 471.61)	.42	0.12	
		1 month	0.05 (–0.33 to 0.43)	0.07 (1, 471.61)	.79	0.06	
	**Accomplishment**						
		10 days	0.20 (–0.24 to 0.64)	0.81 (1, 426.53)	.37	0.12	
		1 month	0.16 (–0.29 to 0.61)	0.51 (1, 449.81)	.48	0.13	
	**Positive emotion**						
		10 days	0.28 (–0.13 to 0.69)	1.81 (1, 395.19)	.18	0.18	
		1 month	–0.05 (–0.47 to 0.37)	0.05 (1, 418.77)	.82	0.01	
	**Engagement**						
		10 days	0.25 (–0.16 to 0.65)	1.42 (1, 472.20)	.23	0.16	
		1 month	0.13 (–0.28 to 0.55)	0.39 (1, 495.92)	.53	0.08	
	**Meaning**						
		10 days	0.18 (–0.31 to 0.66)	0.52 (1, 398.06)	.47	0.10	
		1 month	0.08 (–0.41 to 0.57)	0.10 (1, 416.30)	.76	0.07	
	**Relationships**						
		10 days	–0.13 (–0.57 to 0.31)	0.33 (1, 392.13)	.57	0.05	
		1 month	–0.08 (–0.53 to 0.37)	0.11 (1, 412.73)	.74	0.03	
	**Negative emotion**						
		10 days	0.04 (–0.1 to 0.77)	0.02 (1, 444.16)	.88	0.03	
		1 month	0.05 (–0.41 to 0.51)	0.05 (1, 467.78)	.83	0.01	
	**Health**						
		10 days	–0.01 (–0.47 to 0.46)	<0.001 (1, 404.78)	.98	0.03	
		1 month	–0.13 (–0.6 to 0.35)	0.28 (1, 424.48)	.60	0.01	
	**Loneliness**						
		10 days	0.10 (–0.48 to 0.69)	0.12 (1, 486.60)	.73	0.07	
		1 month	<0.01 (–0.6 to 0.59)	<0.001 (1, 511.24)	.99	0.01	

^a^Mdiff: mean difference.

^b^SUPPH: Strategies Used by People to Promote Health.

^c^eTAP: e-Therapy Attitude and Process Questionnaire.

^d^SCBI: Self-Care Behaviors Inventory.

^e^MHLS: Mental Health Literacy Scale.

^f^PHQ-9: Patient Health Questionnaire.

^g^GAD-7: Generalized Anxiety Disorder Assessment–7.

^h^MAAS: Mindful Attention Awareness Scale.

^i^PERMA: Positive Emotion, Engagement, Relationships, Meaning, and Accomplishment.

#### Self-Care Self-Efficacy

Results revealed no significant difference in self-care self-efficacy (SUPPH) comparing the chatbot and the waitlist control conditions. For the stress reduction subscale, the main effect of time was significant (*F*_2,437.03_=3.70, *P*=.03). Post hoc pairwise comparisons indicated a significant increase in mean scores from baseline to 1-month follow-up (mean difference 0.10, 95% CI 0.01-0.20; *P*=.03) after Bonferroni’s adjustments. However, the time × group interaction was not significant (*F*_2,436.95_=0.36, *P*=.70) and the main effect of group was also not significant (*F*_1,279.55_=0.77, *P*=.38) implying nondifferential change patterns across the 2 groups with their self-efficacy on stress reduction improved over time.

For the positive attitude subscale, no significant time × group interaction effects were observed (*F*_2,392.26_=1.14, *P*=.32). The effects of time (*F*_2,392.34_=0.40, *P*=.67) or group (*F*_1,282.68_=1.22, *P*=.27) were also not significant. Together, the results suggested that the chatbots offered limited effects, with small effect sizes (Cohen *d<*0.18) on self-care self-efficacy among our participants.

#### Behavioral Intention

The time × group interaction effect was significant (*F*_2,379.74_=15.02, *P*<.001). Both groups obtained a significant simple effect of time (chatbot: *F*_2,348.08_=11.99, *P*<.001; control: *F*_2,344.29_=8.49, *P*<.001), but pointing to opposite directions after the 10 days. In the chatbot group, participants’ intention to exercise self-care using digital means increased significantly from baseline to 10 days, with a small-to-moderate effect size (mean difference 0.51, 95% CI 0.16-0.85; *P*=.001; Cohen *d=*0.31). In the control group, a significant drop in participants’ intention from baseline to 10 days was observed (mean difference –0.53, 95% CI –0.86 to –0.21; *P*=.001; Cohen *d=*–0.35). The group difference was significant at 10 days (mean difference 0.88, 0.49-1.28; *P*<.001).

Later at 1-month follow-up, participants’ intention in the chatbot group returned to the baseline as observed in the nonsignificant baseline to 1-month difference (*P*=.93), whereas in the control group, the decrease in behavioral intention remained to be significant (mean difference –0.53, 95% CI –0.91 to –0.14; *P*=.003; Cohen *d=*–0.32). While the chatbots boosted one’s intention to take self-care actions in the short term, the assignment to a waitlist control group appeared to have a negative impact on people’s self-care intention.

#### Self-Care Behavioral Uptake

The time × group interaction effect was significant (*F*_2,369.45_=4.87, *P*=.008). Post hoc pairwise comparisons showed that the simple time effect was only significant in the chatbot group, (*F*_2,340.20_=8.89, *P*<.001) in which self-care behaviors significantly increased from baseline to 10 days (mean difference 0.19, 95% CI 0.08-0.30; *P*<.001; Cohen *d=*0.36), and from baseline to 1 month (mean difference 0.14, 95% CI 0.01-0.26; *P*=.03; Cohen *d=*0.26). In the control group, such improvement was not observed, suggesting that the chatbots were able to enhance the uptake of self-care behaviors, and the effect was sustained at 1-month follow-up.

#### Mental Health Literacy

A significant time × group interaction effect on mental health literacy was found (*F*_2,423.57_=4.27, *P*=.02). Post hoc tests showed that the simple time effect was significant in both groups (chatbot: *F*_2,396.81_=15.51, *P*<.001; waitlist: *F*_2,412.88_=3.52, *P*=.03). In the chatbot group, mental health literacy increased significantly from baseline to 10 days (mean difference 0.25, 95% CI 0.14-0.35; *P*<.001; Cohen *d=*0.45). The effect from baseline to 1 month was also significant (mean difference 0.16, 95% CI 0.05-0.27; *P*=.002; Cohen *d=*0.24). In the control group, improvement from baseline to 10 days was not significant (*P*=.34). However, significant improvement was observed from baseline to 1-month follow-up (mean difference 0.11, 95% CI 0.01-0.21; *P*=.03; Cohen *d=*0.22). The group difference was significant at 10 days (mean difference 0.16, 95% CI 0.02-0.31; *P*=.03; Cohen *d=*0.27). The chatbots were able to improve participants’ mental health literacy to a greater extent.

### Effects on Secondary Outcomes

**Figure 4:**
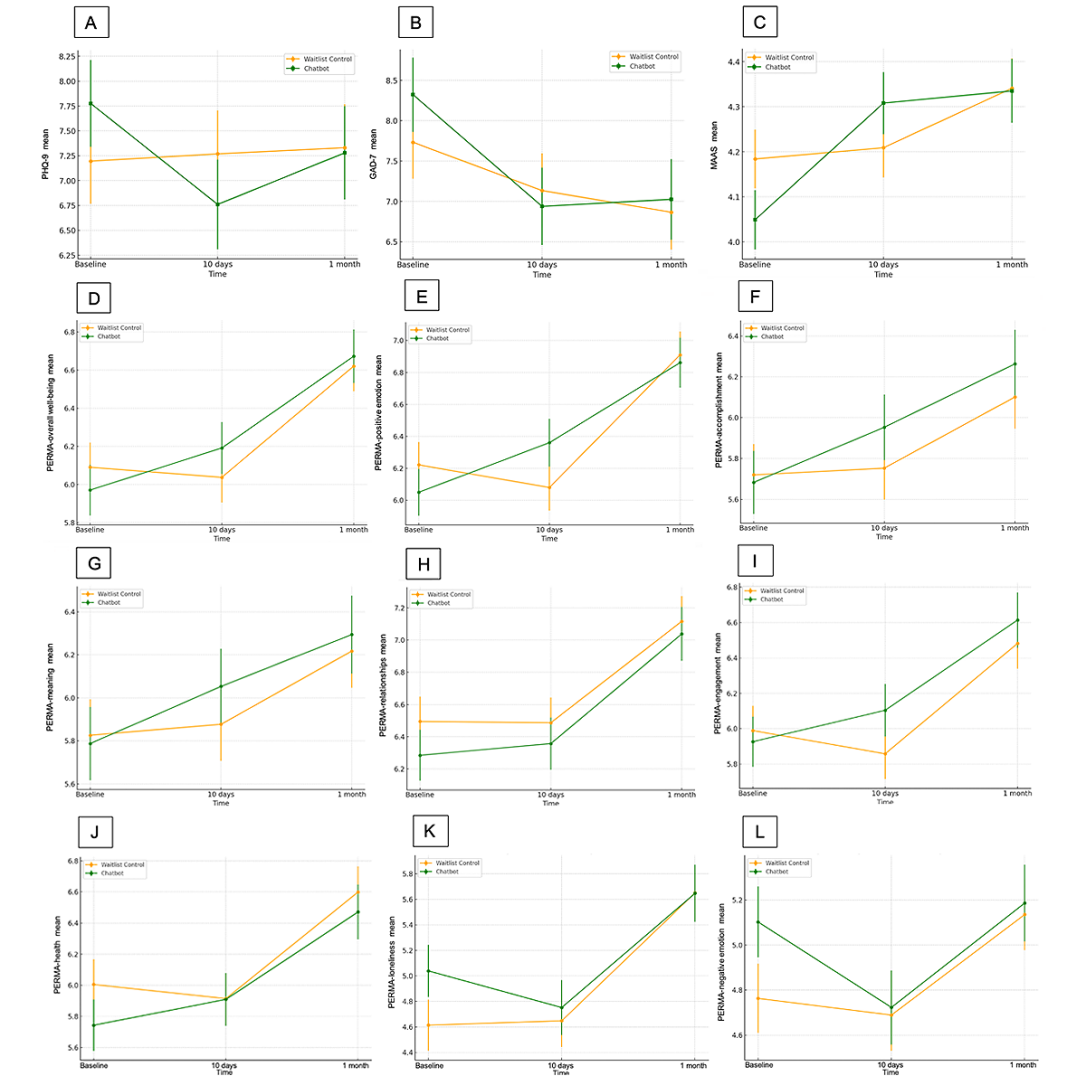
Changes in secondary outcomes over time by condition. (A) Changes in Patient Health Questionnaire–9 (PHQ-9), (B) changes in Generalized Anxiety Disorder Assessment–7 (GAD-7), (C) changes in Mindful Attention Awareness Scale (MAAS), (D) changes in Positive Emotion, Engagement, Relationships, Meaning, and Accomplishment profiler (PERMA)-overall well-being, (E) changes in PERMA-positive emotion, (F) changes in PERMA-accomplishment, (G) changes in PERMA-meaning, (H) changes in PERMA-relationships, (I) changes in PERMA-engagement, (J) changes in PERMA-health, (K) changes in PERMA-loneliness, (L) changes in PERMA-negative emotion.

Figure 4 shows the changes in the secondary outcomes overtime by condition graphically.

#### Depressive and Anxiety Symptoms

A significant time × group interaction effect was obtained for depressive symptoms (*F*_2, 335.12_=3.22, *P*=.04) but not anxiety symptoms (*F*_2,358.21_=1.12, *P*=.33). Post hoc comparisons showed that the improvement on depressive symptoms was only significant in the chatbot group (*F*_2,308.75_=5.44, *P*=.005), in which depressive symptoms reduced significantly from baseline to 10 days with a small-to-moderate effect (mean difference –1.02, 95% CI –1.77 to –0.26; *P*=.004; Cohen *d=*–0.26). However, at 1-month follow-up, depressive symptoms returned to baseline level (*P*=.600). For anxiety symptoms, significant time effect was observed in the chatbot group after Bonferroni adjustment (*F*_2,326.13_=7.52, *P*<.001). Anxiety symptoms reduced significantly from baseline to 10 days (mean difference –1.38, 95% CI –2.30 to –.47; *P*<.001; Cohen *d=*–0.31), and to 1 month (mean difference –1.30, 95% CI –2.34 to –0.25; *P*=.009; Cohen *d=*–0.31). However, this magnitude of improvement did not differ significantly from the waitlist control.

#### Mindfulness

The 2 conditions differed on their effects on participants’ mindfulness level as indicated by the significant interaction (*F*_2,380.83_=5.85, *P*=.003). Both conditions significantly improved mindful awareness over time (chatbot: *F*_2,351.59_=18.58, *P*<.001; waitlist: *F*_2,363.30_=5.92, *P*=.003). Post hoc pairwise comparisons showed that the mean score of Mindful Attention Awareness Scale in the chatbot condition increased significantly from baseline to 10 days (mean difference 0.26, 95% CI 0.14-0.38; *P*<.001; Cohen *d=*0.37), but this increment was not apparent in the waitlist group (*P*>.99). However, from baseline to 1 month both groups increased significantly on their mindfulness levels (chatbot: mean difference 0.29, 95% CI 0.16-0.42, *P*<.001, Cohen *d=*0.47; waitlist: mean difference 0.16, 95% CI 0.04-0.28, *P*=.005, Cohen *d=*0.22).

#### Mental Well-Being

The chatbot participants generally improved their overall mental well-being and most of the well-being dimensions. However, the differences were not large enough to differentiate between the waitlist control participants. Significant time × group interactions were found only on the positive emotion subscale (*F*_2,328.65_=7.08, *P*<.001). Post hoc tests revealed significant time effects on positive emotion in both groups (chatbot: *F*_2,304.49_=22.54, *P*<.001; control: *F*_2,302.39_=40.97, *P*<.001). Pairwise comparisons showed significant improvement from baseline to 10 days only in the chatbot group (mean difference 0.31, 95% CI 0.08-0.54; *P*=.004; Cohen *d=*0.28), not in the control group (*P*=.35). From baseline to 1-month follow-up, both groups improved significantly (chatbot: mean difference 0.81, 95% CI 0.52-1.11, *P*<.001, Cohen *d=*0.55; control: mean difference 0.69, 95% CI 0.42-0.96, *P*<.001, Cohen *d=*0.42).

### Sensitivity Tests

Results of the per-protocol analysis revealed the same pattern of results on primary outcomes as the primary analysis. Slight discrepancies were observed in the secondary outcomes of overall well-being. The time × group interactions were significant in the sensitivity test (*F*_2,295.27_=3.09, *P*=.047). Following up on this significant interaction, the pattern was identical to that of positive emotions noted earlier. Improvement from baseline to 10 days was only significant in the chatbot group (mean difference 0.25, 95% CI 0.04-0.46; *P*=.02) not in the control group (*P*>.99). Furthermore, later both groups improved significantly at 1 month (chatbot: mean difference 0.71, 95% CI 0.46-0.95, *P*<.001; control: mean difference 0.54, 95% CI 0.33-0.76, *P*<.001). Table 6 outlines the result table comparing intention-to-treat and per-protocol analysis.

**Table 6:** Results of primary analysis (intention-to-treat) and sensitivity analyses.

Variable (measurement)		ITT^a^					PP^b^					
		Group × time interaction, *F* test (*df*)		*P* value			Group × time interaction, *F* test (*df*)		*P* value			
**Self-care self-efficacy (SUPPH^c^)**												
	Stress reduction		0.36 (2, 436.95)		.70	0.25 (2, 379.96)		.78				
	Positive attitudes		1.14 (2, 392.26)		.32	1.18 (2, 330.62)		.31				
Behavioral intention (eTAP^d^)		15.02 (2, 379.74)		<.001			16.19 (2, 312.28)		<.001			
Self-care behaviors (SCBI^e^)		4.87 (2, 369.45)		.008			3.26 (2, 314.83)		.04			
Mental health literacy (MHLS^f^)		4.27 (2, 423.57)		.02			3.06 (2, 365.91)		.048			
Depressive symptoms (PHQ-9^g^)		3.22 (2, 335.12)		.04			3.36 (2, 287.28)		.04			
Anxiety symptoms (GAD-7^h^)		1.12 (2, 358.21)		.33			0.59 (2, 314.20)		.56			
Mindfulness (MAAS^i^)		5.85 (2, 380.83)		.003			5.31 (2, 339.54)		.005			
**Well-being (PERMA^j^)**												
	Overall well-being		2.97 (2, 335.76)		.05	3.09 (2, 295.27)		.047				
	Accomplishment		1.13 (2, 354.24)		.32	0.94 (2, 305.05)		.39				
	Positive emotion		7.08 (2, 328.65)		<.001	6.47 (2, 280.32)		.002				
	Engagement		1.65 (2, 391.66)		.19	1.90 (2, 336.54)		.15				
	Meaning		0.85 (2, 386.93)		.43	1.15 (2, 352.35)		.32				
	Relationships		0.32 (2, 354.54)		.72	0.25 (2, 312.62)		.78				
	Negative emotion		1.81 (2, 399.45)		.17	1.42 (2, 343.62)		.24				
	Health		1.25 (2, 374.89)		.29	1.59 (2, 324.49)		.21				
	Loneliness		1.40 (2, 390.58)		.25	1.12 (2, 334.05)		.33				

^a^ITT: intention-to-treat.

^b^PP: per-protocol.

^c^SUPPH: Strategies Used by People to Promote Health.

^d^eTAP: e-Therapy Attitude and Process Questionnaire.

^e^SCBI: Self-Care Behaviors Inventory.

^f^MHLS: Mental Health Literacy Scale.

^g^PHQ-9: Patient Health Questionnaire.

^h^GAD-7: Generalized Anxiety Disorder Assessment–7.

^i^MAAS: Mindful Attention Awareness Scale.

^j^PERMA: Positive Emotion, Engagement, Relationships, Meaning, and Accomplishment.

## Discussion

### Principal Findings

This study evaluated the effectiveness of topic-based chatbots on self-care efficacy, the intention and actual uptake of self-care behaviors, and mental health literacy, alongside associated mental health outcomes, compared to a waitlist control condition. Our findings align with previous research showing immediate improvements, with small-to-medium effect sizes, in mental health outcomes [16-19]. Our primary hypothesis was partially supported as the chatbots did not perform better than the waitlist control on users’ self-care efficacy but significantly promoted their intention and mental health literacy in the short term.

We attribute these findings to the nature of the guidance provided by the chatbots. The chatbots excel at delivering clear information surrounding mental health and actionable steps that users can follow immediately, such as mindful breathing and relaxation exercises. These steps were straightforward and easy to implement, requiring minimal effort. The information presented quickly boosted participants’ literacy. The guidance on short exercises enhances users’ willingness to try out these self-care actions and thus increases their behavioral intention in the short term.

However, enhancing self-efficacy involves more than just doing the practices; it requires building confidence through personalized, reflective, and emotionally supportive interactions [18]. Rather than allowing free-text entry, our rule-based chatbots were developed with predefined, hardcoded responses. While this approach provides structured and predictable interactions, it lacks the flexibility and responsiveness required to address the dynamic nature of mental health concerns and provide personalized reinforcement. The preset multiple-choice type of options also limits users’ depth of reflectivity of their practice experiences necessary for developing self-efficacy. The short 7-day duration of the chatbot intervention may also be insufficient for users to internalize new skills and gain confidence in managing their mental health independently.

The nonsignificant findings regarding the secondary mental health outcomes were disappointing. However, this is understandable as we did not restrict control participants from using other means to take care of their mental health during the study period. In this digital era, people have all sorts of channels to access mental health promotion tools, be it apps, websites, or even joining other intervention studies. This may explain why we observed a sharp improvement among our control participants at the 1-month mark as they may have started exploring other alternatives. We regret for not administrating an exit survey to confirm this claim, future trials should include this to gain a better understanding of attrition.

Among the few studies that discussed the chatbot’s effect on well-being, minimally small differences between the intervention and waitlist groups were obtained [53,54]. Similarly, we found small differences between the groups on most well-being domains. The chatbots were more able to enhance users’ positive emotions. We suspected the chatbot’s engaging features provided satisfying experiences and positive reinforcement that are effective toward their mental health, at least in the short term.

We acknowledge the rapid advancements in chatbot technology; at the time when this study was planned, Gen AI chatbots powered by large language models (eg, OpenAI’s ChatGPT, Microsoft’s Azure) were not as accessible as they are nowadays. Our chatbots were designed with decision trees following the convention back then. Previous research on rule-based chatbots reported negative experiences and perceptions, such as being repetitive, boring, and unfunctional, making users feel bothered [17,55,56]. These issues may also apply to this study. Unlike rigid rule-based scripts, Gen AI chatbots learn from extensive datasets, refining responses in real-time. They can interpret users’ inputs, adapt to particular context, and deliver personalized content to improve user engagement. However, Gen AI chatbots are not free from challenges. They could generate responses that seem convincing but are factually incorrect, that is, “hallucinations” [57]. While these models hold the potential to expand mental health support and reach underserved populations, the need for transparent design, rigorous evaluation, and appropriate clinical safeguards cannot be understated [58].

In addition, this study recruited adults aged ≥18 years who possessed proficiency in using computers and comprehension of language. Recruitment was done primarily through web-based platforms and snowballing, contributing to a selective sample of individuals with better eHealth literacy, greater interest in mental well-being, and a higher acceptance to engage with mental health chatbots. This aligns with our attrition analysis, which found that people who stayed had better baseline mental health. Therefore these results cannot be generalized to the broader population of individuals who might not be as technology-savvy and mentally well. Future studies could investigate the feasibility of these chatbots in other populations, such as older adults, adolescents, and people in rural areas.

Despite these limitations, this study is one of the few studies that tested the effectiveness of chatbots on self-care intentions and behaviors, using a rigorous, adequately powered randomized controlled trial design. There is an abundance of mental health self-care tools available in the market, offering a wide array of options for consumers. While this proliferation of resources broadens the choices for individuals seeking mental support, it also creates risks. Many of these tools are not empirically tested or are misinformed [59-61]. The topic-based chatbots used in this study were designed with evidence-based approaches (eg, cognitive behavioral therapy and mindfulness-based intervention), and the composition of the content was developed and supervised by clinical psychologists. There was no adverse event reported during the trial. In this sense, our chatbots are empirically supported and safe to use.

### Conclusions

In conclusion, this study provides empirical support for the preventive potential of rule-based chatbots in enhancing mental health literacy and increasing the immediate intention to practice self-care. However, their ability to promote self-care self-efficacy and long-term mental health improvements is limited. The chatbots’ structured, evidence-based content likely contributed to the improvements in mental health literacy and short-term behavioral intentions, while the lack of personalization may explain the limited impact on self-efficacy. These findings highlight the need for more advanced and personalized chatbot interventions to leverage technology effectively for mental health promotion. Nonetheless, these topic-based mental health chatbots may help bridge the gap between the high demand for mental health services and scarce resources by initiating people’s self-care efforts. Future research should continue to empirically test Gen AI chatbots, explore the specific mechanisms through which chatbots cultivate personal assets, examine their effectiveness in diverse populations, and explore hybrid approaches combining rule-based and Gen AI systems.

## Data Availability

The datasets generated or analyzed during this study are available from the corresponding author on reasonable request.

## Appendix

Multimedia Appendix 1 CONSORT-eHEALTH checklist (V 1.6.1).

## Abbreviations

CONSORT: Consolidated Standards of Reporting Trials
eTAP: e-Therapy Attitude and Process Questionnaire
GAD-7: Generalized Anxiety Disorder Assessment–7
Gen AI: generative artificial intelligence
LMM: linear mixed model
PERMA: Positive Emotion, Engagement, Relationships, Meaning, and Accomplishment
PHQ-9: Patient Health Questionnaire–9
RCT: randomized controlled trial
SCBI: Self-Care Behaviors Inventory
SUPPH: Strategies Used by People to Promote Health
